# Adventitial Tertiary Lymphoid Organs as Potential Source of MicroRNA Biomarkers for Abdominal Aortic Aneurysm

**DOI:** 10.3390/ijms160511276

**Published:** 2015-05-18

**Authors:** Rafaelle Spear, Ludovic Boytard, Renaud Blervaque, Maggy Chwastyniak, David Hot, Jonathan Vanhoutte, Bart Staels, Yves Lemoine, Nicolas Lamblin, François-René Pruvot, Stephan Haulon, Philippe Amouyel, Florence Pinet

**Affiliations:** 1 National Institute of Health and Medical Research (INSERM) U1167, Lille Pasteur Institute, Lille North of France University, F-59000 Lille, France; E-Mails: rafaelle.spear@pasteur-lille.fr (R.S.); ludovic.boytard@laposte.net (L.B.); maggy.chwastyniak@pasteur-lille.fr (M.C.); nicolas.lamblin@chru-lille.fr (N.L.); philippe.amouyel@pasteur-lille.fr (P.A.); 2 INSERM U1019, National Center of Scientific Research (CNRS) Join Research Unit (UMR) 8204, Lille Pasteur Institute, Lille North of France University, F-59000 Lille, France; E-Mails: renaudblervaque@hotmail.com (R.B.); david.hot@pasteur-lille.fr (D.H.); yves.lemoine@pasteur-lille.fr (Y.L.); 3 INSERM U1011, Lille Pasteur Institute, Lille North of France University, F-59000 Lille, France; E-Mails: jonathan.vanhoutte@pasteur-lille.fr (J.V.); bart.staels@pasteur-lille.fr (B.S.); 4 Lille Regional University Hospital, F-59037 Lille, France; E-Mail: francois-rene.pruvot@chru-lille.fr; 5 INSERM U1008, Lille North of France University, Lille Regional University Hospital, F-59000 Lille, France; E-Mail: stephan.haulon@chru-lille.fr

**Keywords:** abdominal aortic aneurysm, adventitial tertiary lymphoid organs, microRNA, laser microdissection, quantitative RT-PCR

## Abstract

Abdominal aortic aneurysm (AAA) is an inflammatory disease associated with marked changes in the cellular composition of the aortic wall. This study aims to identify microRNA (miRNA) expression in aneurysmal inflammatory cells isolated by laser microdissection from human tissue samples. The distribution of inflammatory cells (neutrophils, B and T lymphocytes, mast cells) was evaluated in human AAA biopsies. We observed in half of the samples that adventitial tertiary lymphoid organs (ATLOs) with a thickness from 0.5 to 2 mm were located exclusively in the adventitia. Out of the 850 miRNA that were screened by microarray in isolated ATLOs (*n* = 2), 164 miRNAs were detected in ATLOs. The three miRNAs (miR-15a-3p, miR-30a-5p and miR-489-3p) with the highest expression levels were chosen and their expression quantified by RT-PCR in isolated ATLOs (*n* = 4), M1 (*n* = 2) and M2 macrophages (*n* = 2) and entire aneurysmal biopsies (*n* = 3). Except for the miR-30a-5p, a similar modulation was found in ATLOs and the two subtypes of macrophages. The modulated miRNAs were then evaluated in the plasma of AAA patients for their potential as AAA biomarkers. Our data emphasize the potential of miR-15a-3p and miR-30a-5p as biomarkers of AAA but also as triggers of ATLO evolution. Further investigations will be required to evaluate their targets in order to better understand AAA pathophysiology.

## 1. Introduction

Abdominal aortic aneurysm (AAA) is a complex vascular disease and represents a public health care problem, responsible for more than 12,217 deaths in the United States in 2009 [1]. This high mortality is largely due to the asymptomatic progression before rupture in nearly all AAA patients and, has enhanced the interest for the search for biomarkers that can be detected easily in blood, thus facilitating systemic screening of the population at risk [2].

Deciphering the targets (ribonucleic acids (RNA), microRNA (miRNA), proteins) involved in AAA might help in this search of biomarkers. MiRNAs, which are small non-coding RNAs, have recently been shown to be molecular markers because of their role in transcriptional and posttranscriptional regulation [3]. Their stability in plasma enhances their potential as biomarkers [4]. Recently, five miRNAs (miR-181a*, miR-146a, miR-21, miR-331-3p and miR-204) were shown to be differentially expressed in the human aneurysmal wall when compared to control human aorta [5].

AAA is a complex disease, which is associated with marked changes in the cellular composition of the aortic wall. One change is smooth muscle cell (SMC) apoptosis by cell detachment from the extracellular matrix, resulting in less elasticity and less rigidity of aortic wall [6]. Inflammation is a key player in aneurysmal pathology as inflammatory cells are the major source not only of metalloproteases (MMP) [7] but also of cathepsins [8]. Numerous inflammatory cells are involved in AAA formation and growth [7,9]. Recently, we showed that the two subtypes of macrophages, M1 with proinflammatory properties and M2, with anti-inflammatory properties are distributed differently in the aneurysmal wall [10]. Neutrophils are known to be involved in AAA [11] by recruiting other inflammatory cells [9] or producing MMPs such as MMP9 [12]. Another cell type that might be involved in AAA is mast cells. Indeed, Zhang *et al.*, showed no development of an aneurysm in mast cell-deficient mice [13]. Mast cells are also a source of cytokines that attract other inflammatory cells and then amplify the immune response [14]. Lymphocytes are also implicated in AAA, but their role is less clear. Studies have shown that T lymphocytes were predominant in AAA but that B lymphocytes were also present in the aortic wall [7]. In fact, T and B lymphocytes have been described to be organized in follicle-like structures in murine models of atherosclerosis [15]. Recently, these inflammatory structures named adventitial tertiary lymphoid organs (ATLOs) have been described in murine aneurysm models with a prevalent occurrence in the abdominal aorta [16].

Tertiary lymphoid organs develop at sites of chronic inflammation with antigen stimulation [17]. It was hypothesized that ATLOs may be involved in communication between the intima and the adventitia that results in adventitial inflammation accompanied by tissue destruction as in AAA [18].

The aim of this study was to profile, with microarray technology, miRNAs in ATLOs isolated by laser capture-microddissection (LCM) in order to detect potential targets of AAA not revealed by a hypothesis-driven approach [2]. We therefore first analyzed the specific distribution of inflammatory cells (neutrophils, B and T lymphocytes and mast cells) as we did previously for the two subtypes of macrophages [10] in the different layers of AAA. The expression of the selected miRNAs detected in ATLOs was then evaluated by quantitative RT-PCR (qRT-PCR) in LCM-isolated ATLOs, M1 and M2 macrophages as well as in entire aneurysmal and control aorta samples. We then evaluated their plasma expression in patients with AAA compared to patients with no AAA [19].

## 2. Results and Discussion

Although aneurysms may develop along the entire length of the aorta, they are at least five times more prevalent in the abdomen than in the thorax [20]. The aneurysmal aortic wall is a complex tissue composed of different cell types (e.g., inflammatory cells and SMCs) at different times during the course of the disease [21]. Inflammation is not only associated with the clinical presence of AAA, but also plays a key role in the pathogenesis of the disease [22] and has recently been related to aortic thrombus formation [23]. This study therefore aimed first to determine the distribution of inflammatory cells in the different layers of human AAA in order to determine the presence of B/T cell aggregates, which have been described as precursor of ATLOs [16].

### 2.1. Distribution of Inflammatory Cells in the Human Aneurysmal Aortic Wall

**Figure 1:**
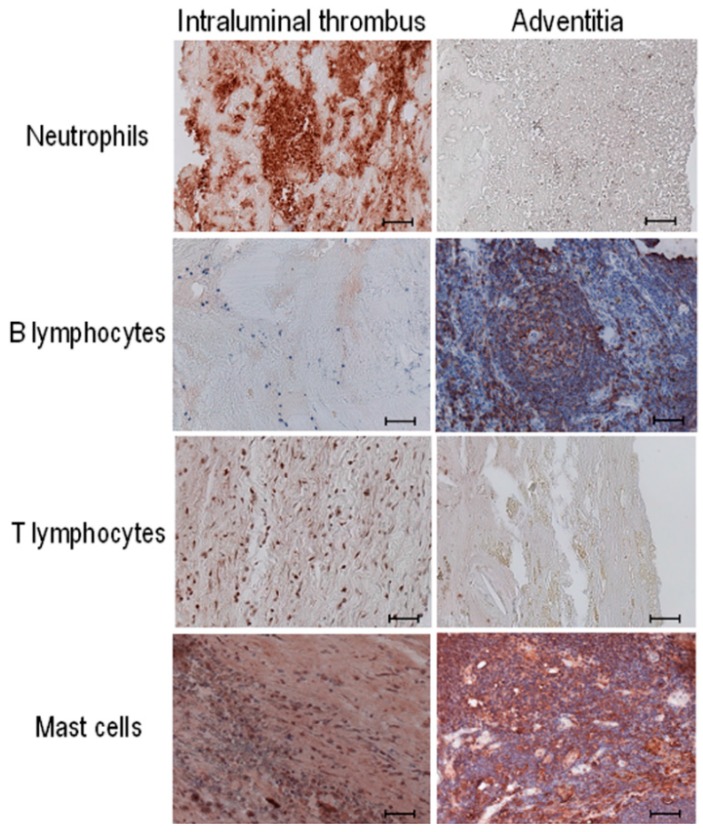
Distribution of inflammatory cells in AAA biopsies by immunostaining. Stained cells were analyzed in the intraluminal thrombus (**left** panels) and adventitia (**right** panels) of the aneurysmal aortic wall. The inflammatory cells visualized are neutrophils (anti-CD66e), B lymphocytes (anti-CD20), T lymphocytes (anti-CD3) and mast cells (anti-mast cell tryptase). Immunostaining analysis was performed in every collected AAA sample (*n* = 20). Scale bar: 50 µm.

We looked for the presence and distribution of the inflammatory cells in human AAA, bearing in mind however that surgical specimens of human AAA collected from patients undergoing open surgery represent an advanced stage of the disease. Macroscopic analysis was systematically performed to orientate aneurysmal biopsies. Figure 1 depicts the distribution of the inflammatory cells towards the aneurysmal wall, namely neutrophils (CD66e staining), B lymphocytes (CD20 staining), T lymphocytes (CD3 staining) and mast cells (mast cell tryptase staining). We found neutrophils to be present in the intraluminal thrombus and in the adventitia of the AAA wall, as previously described by Houard *et al.* [9]. We observed T lymphocytes in the intraluminal thrombus, but only in a few AAA samples. In contrast, B lymphocytes were detected predominantly in the adventitia.

Our results show a specific distribution of inflammatory cells towards the aneurysmal aortic wall. Not every AAA tissue sample contains every type of inflammatory cell: The individual AAA samples are heterogeneous from patient to patient and vary according to disease complexity. As expected, we observed no inflammatory cells in the media, as reviewed by Michel *et al*. [24]. In the adventitia, B lymphocytes and mast cells predominated, together with the proinflammatory CD68^+^MR^−^ (Mannose Receptor) macrophage subtype as previously shown [10] and neutrophils and T lymphocytes predominated, together with the anti-inflammatory subtype CD68^+^MR^+^ macrophages as previously shown in the intraluminal thrombus [10].

### 2.2. Presence of Adventitial Tertiary Lymphoid Organs

**Figure 2:**
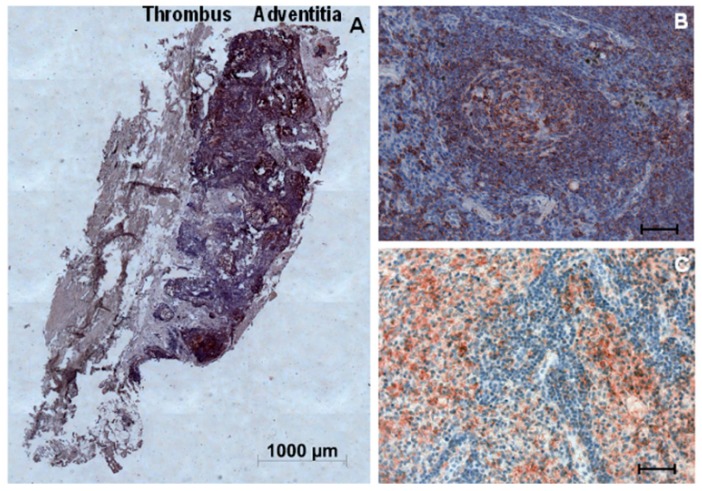
Characterization of adventitial tertiary lymphoid organ (ATLO) in AAA biopsies by immunostaining. (**A**) Representative AAA section with intraluminal thrombus (**left**) and adventitia (**right**); (**B**) B lymphocytes were stained with anti-CD20 antibody; and (**C**) Mast cells were stained with anti-mast cell tryptase antibody. Scale bar: 1000 µm in (**A**) and 50 µm in (**B**,**C**).

Interestingly, in seven out of twenty samples tested, we could observe cells condensed into compacted structures occupying the entire adventitial layer, accounting for a major part of the aneurysm wall (Figure 2A). These structures described as ATLOs [25] were mainly composed of B lymphocytes (Figure 2B) and mast cells (Figure 2C).

The relative quantification of the thickness and area of ATLO showed that thickness ranged between 0.5 and 2 mm and accounted for as much as 9% to 42% of the total aneurysmal aortic wall area (Table 1).

**Table 1:** Quantification of adventitial tertiary lymphoid organ (ATLO) thickness and area in the human aneurysmal aorta.

Sample Number	Thickness (mm)		ATLO (%)	Area (mm²)		ATLO (%)
	ATLO	Total		ATLO	Total	
6	2.1	6	35.0	3.9	25.0	15.6
8	1.4	3.3	42.4	3.5	12.8	27.3
11	0.7	4.5	15.6	0.5	30.5	1.6
13	0.5	5.5	9.1	0.4	20.3	2.0
15	0.5	2.4	20.8	0.4	62.1	0.6
19	2.2	5.8	37.9	5.3	29.5	18.0
20	1.7	4.4	38.6	5.1	19.6	26.0
Mean	1.3	4.6	28.5	2.7	28.5	9.6

ATLO: Adventitial tertiary lymphoid organ.

ATLOs were found in seven out of the 20 AAA biopsies studied, confirming previous observation [25]. The interaction hypothesized between ATLOs, inflammatory cells and the medial SMCs enhance their potential to be a source of biomarkers of AAA [18]. ATLOs are associated with leukocyte infiltration, including CD68^+^ cells of the abdominal aorta and have been described to emerge in adult in response to persistent inflammation.

The great diversity of the aneurysmal aortic wall, in which inflammatory cell types are not present in the same aneurysmal area, confirms the complexity of AAA pathology. The importance of studying isolated cells from AAA instead of whole tissue analysis is increased by the presence of ATLO and the cell diversity of the AAA wall, in which inflammatory cells can have opposite functions, as proinflammatory (CD68^+^MR^−^) and anti-inflammatory (CD68^+^MR^+^) macrophages [26]. In fact, Sho *et al*. [27], reported that analysis of isolated cells highlighted specific targets of interest that are hidden on whole-aorta tissue.

### 2.3. Profile and Quantification of miRNAs in Adventitial Tertiary Lymphoid Organs (ATLOs) Isolated by Laser Capture Microdissection

Areas rich in ATLOs were located by immunostaining with anti-CD20 antibody and then isolated by LCM on two different aneurysmal tissue samples. We isolated an average of 13.5 (11–16) mm^2^ of ATLOs corresponding to 1100 ng of RNA with an average RNA Integrity Number of 4.9 [2.8–6.9].

ATLOs isolated by LCM were screened for 850 human miRNAs using a microarray-based assay. After normalization, 164 miRNAs were considered present in the ATLOs samples (Table 2).

**Table 2:** List of miRNAs detected in laser capture-microdissection (LCM)-isolated ATLO samples.

miRNA	Normalized Mean Values ± SD [Sample 1–Sample 2]	miRNA	Normalized Mean Values ± SD [Sample 1–Sample 2]
let-7b-3p	4.73 ± 2.21 [6.30–3.17]	miR-320b	8.93 ± 0.22 [9.08–8.77]
let-7e-5p	5.24 ± 0.032 [5.22–5.27]	miR-325	5.15 ± 0.72 [4.64–5.66]
let-7f-5p	5.24 ± 1.49 [4.18–6.29]	miR-330-3p	9.23 ± 2.37 [7.56–10.91]
let-7f-2-3p	7.90 ± 0.10 [7.83–7.97]	miR-337-3p	2.70 ± 1.19 [1.86–3.54]
miR-101-3p	5.71 ± 0.37 [5.97–5.45]	miR-337-5p	2.70 ± 1.139 [1.86–3.54]
miR-106a-3p	10.56 ± 0.99 [11.26–9.86]	miR-339-5p	3.74 ± 2.02 [5.17–2.31]
miR-107	8.38 ± 0.11 [8.31–8.46]	miR-33a-3p	3.13 ± 1.58 [2.02–4.24]
miR-1179	4.42 ± 0.48 [4.76–4.08]	miR-340-5p	4.22 ± 2.40 [2.52–5.92]
miR-1181	3.79 ± 0.49 [4.14–3.44]	miR-342-3p	5.72 ± 0.19 [5.58–5.85]
miR-1183	4.92 ± 1.85 [3.61–6.22]	miR-345-5p	3.43 ± 1.29 [4.34–2.52]
miR-1201_v15.0	4.73 ± 2.21 [6.29–3.17]	miR-34a-5p	6.41 ± 1.14 [5.61–7.22]
miR-1203	5.72 ± 0.19 [5.58–5.85]	miR-34a-3p	3.62 ± 2.49 [5.38–1.86]
miR-1208	7.05 ± 0.00 [7.05–7.05]	miR-34c-3p	8.40 ± 0.26 [8.58–8.22]
miR-122-5p	3.01 ± 1.82 [4.29–1.72]	miR-362-3p	5.89 ± 1.13 [5.09–6.69]
miR-1224-3p	4.73 ± 2.21 [6.29–3.17]	miR-369-5p	10.56 ± 0.99 [11.26–9.86]
miR-1224-5p	3.19 ± 1.92 [4.55–1.84]	miR-370-3p	2.55 ± 2.73 [4.48–0.62]
miR-1226-3p	1.59 ± 0.91 [2.23–0.95]	miR-374a-3p	7.66 ± 0.03 [7.68–7.64]
miR-1234-3p	4.73 ± 2.21 [6.29–3.17]	miR-379-3p	3.07 ± 1.37 [4.05–2.10]
miR-1238-3p	3.07 ± 1.37 [4.05–2.10]	miR-409-5p	1.59 ± 0.91 [2.23–0.95]
miR-1250-5p	7.48 ± 0.77 [8.02–6.93]	miR-431-3p	5.30 ± 1.02 [4.58–6.03]
miR-1251-5p	4.92 ± 1.85 [3.61–6.22]	miR-432-3p	1.59 ± 0.91 [2.23–0.95]
miR-1252-5p	4.63 ± 1.54 [3.54–5.72]	miR-451a	4.92 ± 1.85 [3.61–6.22]
miR-125b-1-3p	1.76 ± 2.44 [3.49–0.03]	miR-452-3p	4.79 ± 0.21 [4.94–4.64]
miR-1262	6.13 ± 0.74 [6.65–5.61]	miR-454-5p	5.98 ± 0.18 [5.85–6.10]
miR-1267	4.73 ± 2.21 [6.29–3.17]	miR-483-5p	2.95 ± 2.34 [1.30–4.61]
miR-127-3p	5.71 ± 0.37 [5.97–5.45]	miR-486-5p	6.10 ± 1.24 [6.98–5.22]
miR-1280	1.76 ± 2.44 [3.49–0.03]	miR-487a-3p	4.42 ± 0.48 [4.76–4.08]
miR-1281	5.15 ± 0.72 [4.64–5.66]	miR-487b-3p	7.30 ± 0.79 [7.82–6.74]
miR-1282	3.74 ± 2.02 [5.17–2.31]	miR-488-5p	2.34 ± 1.03 [3.07–1.61]
miR-1290	4.66 ± 0.60 [4.24–5.08]	**miR-489-3p**	**12.42 ± 0.00 [12.42–12.42]**
miR-1294	3.43 ± 0.70 [3.93–2.94]	miR-493-3p	6.52 ± 1.05 [7.26–5.78]
miR-1296-5p	3.53 ± 2.19 [5.08–1.99]	miR-497-5p	5.71 ± 0.37 [5.97–5.45]
miR-1303	5.15 ± 0.72 [4.64–5.66]	miR-504-5p	4.15 ± 0.59 [3.73–4.57]
miR-1307-3p	5.37 ± 0.62 [4.93–5.81]	miR-508-5p	4.22 ± 2.40 [2.52–5.92]
miR-130b-5p	6.12 ± 0.45 [5.80–6.44]	miR-513a-5p	6.26 ± 1.77 [5.00–7.51]
miR-132-5p	4.73 ± 2.21 [6.29–3.17]	miR-517a-3p	8.90 ± 0.34 [8.65–9.14]
miR-1321	5.71 ± 0.376 [5.97–5.45]	miR-517b-3p	10.35 ± 0.26 [10.53–10.16]
miR-1323	7.57 ± 0.63 [7.13–8.02]	miR-518a-5p	4.73 ± 2.21 [6.29–3.17]
miR-136-5p	6.43 ± 0.96 [5.75–7.11]	miR-518c-5p	3.79 ± 0.49 [4.14–3.44]
miR-136-3p	3.91 ± 1.75 [2.68–5.15]	miR-519e-5p	3.07 ± 1.36 [2.11–4.03]
miR-140-3p	3.42 ± 1.83 [2.13–4.72]	miR-522-3p	9.29 ± 0.41 [9.59–9.00]
miR-144-5p	6.87 ± 0.03 [6.89–6.85]	miR-548c-3p	8.38 ± 0.11 [8.31–8.46]
miR-146a-5p	9.65 ± 0.37 [9.92–9.39]	miR-548g-3p	2.70 ± 1.19 [1.86–3.54]
miR-147a	3.92 ± 0.07 [3.98–3.87]	miR-548m	4.66 ± 0.60 [4.24–5.08]
miR-155-3p	6.52 ± 1.05 [7.26–5.78]	miR-548p	4.73 ± 2.21 [6.29–3.17]
**miR-15a-3p**	**10.35 ± 0.26 [10.53–10.16]**	miR-550a-5p	3.92 ± 2.18 [5.46–2.38]
miR-17-3p	0.95 ± 0.77 [1.50–0.41]	miR-551b-3p	2.55 ± 2.73 [4.48–0.62]
miR-181a-5p	7.72 ± 0.04 [7.75–7.70]	miR-552-3p	3.13 ± 1.58 [2.02–4.24]
miR-181a-2-3p	7.54 ± 1.05 [6.80–8.28]	miR-555	3.42 ± 1.89 [2.08–4.75]
miR-183-5p	4.1 ± 1.18 [3.24–4.93]	miR-571	4.73 ± 2.21 [6.29–3.17]
miR-184	6.01 ± 0.45 [5.69–6.33]	miR-573	4.73 ± 2.21 [6.29–3.17]
miR-185-3p	6.87 ± 0.03 [6.89–6.85]	miR-574-5p	8.38 ± 0.27 [8.18–8.57]
miR-186-5p	1.82 ± 0.35 [1.58–2.07]	miR-578	4.64 ± 0.92 [5.29–3.99]
miR-186-3p	2.04 ± 2.62 [3.90–0.19]	miR-584-5p	10.34 ± 1.44 [9.32–11.36]
miR-187-5p	3.74 ± 2.02 [5.17–2.31]	miR-591	5.95 ± 0.77 [5.41–6.50]
miR-18b-5p	8.08 ± 0.00 [8.08–8.08]	miR-592	6.73 ± 0.53 [7.10–6.35]
miR-1912	3.04 ± 0.08 [3.09–2.98]	miR-593-3p	4.76 ± 2.32 [3.12–6.40]
miR-196b-5p	3.42 ± 1.89 [2.08–4.75]	miR-599	5.98 ± 0.12 [5.90–6.07]
miR-197-3p	8.17 ± 1.03 [7.44–8.89]	miR-607	0.95 ± 0.77 [1.50–0.41]
miR-199a-3p	6.01 ± 0.45 [5.69–6.33]	miR-615-3p	2.70 ± 1.19 [1.86–3.54]
miR-200a-5p	4.73 ± 2.21 [6.29–3.17]	miR-616-5p	7.62 ± 0.44 [7.94–7.31]
miR-200c-3p	3.91 ± 1.75 [2.68–5.15]	miR-619-3p	4.73 ± 2.21 [6.29–3.17]
miR-202-3p	1.57 ± 1.087 [2.34–0.80]	miR-621	8.38 ± 0.27 [8.18–8.57]
miR-202-5p	6.87 ± 0.03 [6.89–6.85]	miR-624-5p	9.15 ± 0.47 [8.81–9.48]
miR-205-5p	4.64 ± 0.92 [5.29–3.99]	miR-628-3p	6.26 ± 1.77 [5.00–7.51]
miR-206	5.98 ± 0.18 [5.85–6.10]	miR-637	3.19 ± 1.92 [4.55–1.84]
miR-218-1-3p	3.43 ± 1.29 [4.34–2.52]	miR-642a-5p	6.78 ± 0.27 [6.59–6.98]
miR-218-2-3p	5.02 ± 2.39 [6.72–3.33]	miR-646	4.73 ± 2.21 [6.29–3.17]
miR-220c_v15.0	4.64 ± 0.92 [5.29–3.99]	miR-654-5p	9.29 ± 0.41 [9.59–9.00]
miR-23b-3p	5.02 ± 2.39 [6.72–3.33]	miR-661	3.17 ± 2.65 [5.05–1.30]
miR-26b-5p	9.86 ± 0.24 [10.03–9.69]	miR-663b	4.80 ± 0.12 [4.72–4.89]
miR-27a-5p	8.69 ± 0.06 [8.73–8.65]	miR-758-3p	6.43 ± 0.96 [5.75–7.11]
miR-296-5p	8.93 ± 0.22 [9.08–8.77]	miR-770-5p	4.92 ± 1.85 [3.61–6.22]
miR-29a-3p	3.92 ± 2.18 [5.46–2.38]	miR-802	4.42 ± 0.48 [4.76–4.08]
miR-29c-3p	3.04 ± 0.08 [3.09–2.98]	miR-873-5p	4.22 ± 2.40 [2.52–5.92]
miR-302b-3p	2.57 ± 2.64 [4.44–0.71]	miR-875-5p	3.13 ± 1.58 [2.02–4.24]
miR-302d-5p	4.15 ± 0.59 [3.73–4.57]	miR-877-3p	2.01 ± 0.489 [2.35–1.68]
**miR-30a-5p**	**12.42 ± 0.00 [12.42–12.42]**	miR-891b	7.62 ± 0.44 [7.94–7.31]
miR-30a-3p	3.43 ± 1.29 [4.34–2.52]	miR-922	2.12 ± 2.78 [4.09–0.16]
miR-30c-1-3p	8.09 ± 0.43 [8.39–7.78]	miR-924	2.70 ± 1.19 [1.86–3.54]
miR-32-5p	4.15 ± 0.51 [4.51–3.80]	miR-92b-5p	4.63 ± 1.54 [3.54–5.72]
miR-32-3p	5.34 ± 1.88 [4.01–6.67]	miR-935	9.23 ± 2.37 [7.56–10.91]

The miRNAs selected for further analysis are indicated in bold.

Based on the average of their normalized values, we selected the top three miRNAs with a difference of value <1: miR-15a-3p, miR-30a-5p, miR-489-3p for further analysis by qRT-PCR.

Several studies have suggested that vascular SMC in the media may coordinate the cross talk between intimal atherosclerotic lesions and adventitial inflammation [18]. Recently, M1 macrophages were identified as lymphoid tissue inducer cells in mice [28].

**Figure 3:**
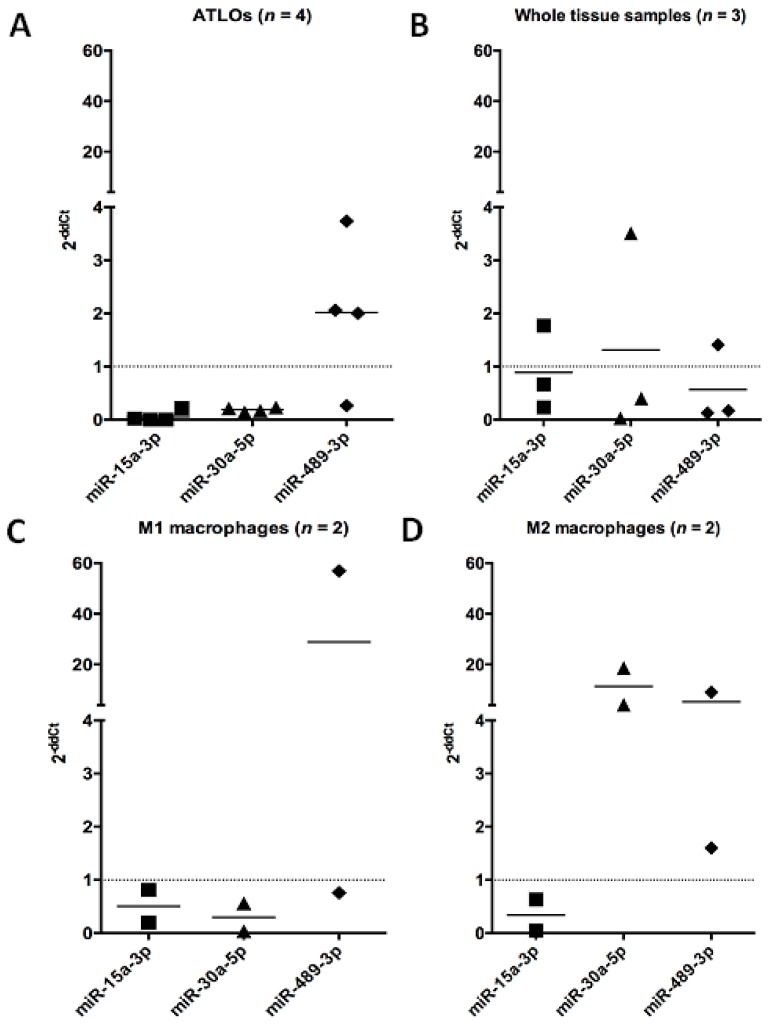
Relative quantification of the three miRNAs selected in LCM-isolated ATLOs (*n* = 4) (A), M1 (*n* = 2) (C), M2 macrophages (*n* = 2) (D) and in whole aortic aneurysmal biopsies (*n* = 3) (B) by qRT-PCR with the −2ΔΔ*C*_t_ method. Control SMC (*n* = 2) and control aorta (*n* = 3) were used as reference for the quantification in LCM-isolated cells and aneurysmal aorta, respectively and RNU6-2 for the calibration. Data are expressed in Log (2^−ΔΔ*C*t^).

The three miRNAs selected from the microarray screening were analyzed by qRT-PCR comparing their expression in LCM-isolated ATLOs to non-aneurysmal SMC. We found that miR-15a-3p (0.1-fold) and miR-30a-5p (0.2-fold) were down-regulated and miR-489-3p up-regulated (2 fold) in ATLOs (Figure 3).

We then compared the expression of the three selected miRNAs in M1 and M2 macrophages isolated by LCM as previously described [10]. We found a similar down-regulation of miR-15a-3p in M1 (0.5-fold) and M2 (0.3-fold) macrophages. MiR-30a-5p was down-regulated (0.3-fold) in M1 macrophages and up-regulated (11.4-fold) in M2 macrophages. Concerning miR-489-3p, we found a similar modulation for M1 (28.8-fold) and M2 (5.4-fold) macrophages. Modulation of miRNAs between ATLOs and M1 macrophages was similar for the three miRNAs, in accordance with the potential of M1 as inducer of lymphoid tissue [28], though these data are preliminary due to the limited number of samples tested. We observed a converse expression of miR-30a-5p in isolated aneurysmal M1 and M2 macrophages. Recently, it was shown that miR-30a-5p with eight others was differently expressed in THP-1 macrophages in response to infectious agent such as *M. tuberculosis* [29] and regulated suppressor of cytokine signaling 3 in ApoE^−/−^ mice, which is implicated in the anti-apoptotic pathway [30]. In contrast, miR-15a-3p was similarly regulated in the isolated aneurysmal cells tested as well in the whole aorta. Mir-15a-3p has been described to be a regulator of angiogenesis through its interaction with Vascular Endothelial Growth Factor [31]. Interestingly, ATLOs require endothelial venules to interact with the media and other inflammatory cells [32], and are triggered by chemokines, which could be regulated in response to the enhanced development of ATLOs [16].

To underline the utility of analyzing microdissected ATLOs, we evaluated the expression of the three selected miRNAs in whole biopsies of aneurysmal and control aorta. The three miRNAs, miR-15a-3p, miR-30a-5p and miR-489-3p, were down-regulated 0.6-, 0.25- and 0.2-fold, respectively (Figure 3).

These latter data confirmed the interest to study individually the cells as shown by the inverse expression of miR-489-3p in the whole aneurysmal aorta. MiR-489-3p was identified in hypertrophic cardiomyocytes but its expression was reduced after angiotensin treatment [33]. Its role and targets in AAA will require further exploration.

To evaluate the 3 miRNAs selected as potential circulating AAA markers, we quantified their plasma levels in patients presenting both atherosclerosis and AAA and in patients with peripheral arterial diseases (PAD) and non-aneurysmal atherosclerosis [19]. AAA patients were significantly older than the PAD controls, other clinical risk factors were similar between the groups (Table 3).

**Table 3:** Risk factors of the study population.

Cardiovascular Risk Factors	AAA ( *n* = 24)	PAD ( *n* = 18)	*p*
Age, years	68.0 ± 6.1	63.3 ± 6.6	0.006
Male gender (%)	24 (100)	18 (100)	ND
Current smoking	4 (17)	4 (22)	0.71
Past smoking	14 (58)	12 (67)	0.75
Hypercholesterolemia	15 (63)	10 (56)	0.75
Diabetes mellitus	4 (17)	5 (28)	0.46
Familial history of CAD	4 (17)	2 (13)	1.0

CAD, coronary disease; ND, not determined.

**Figure 4:**
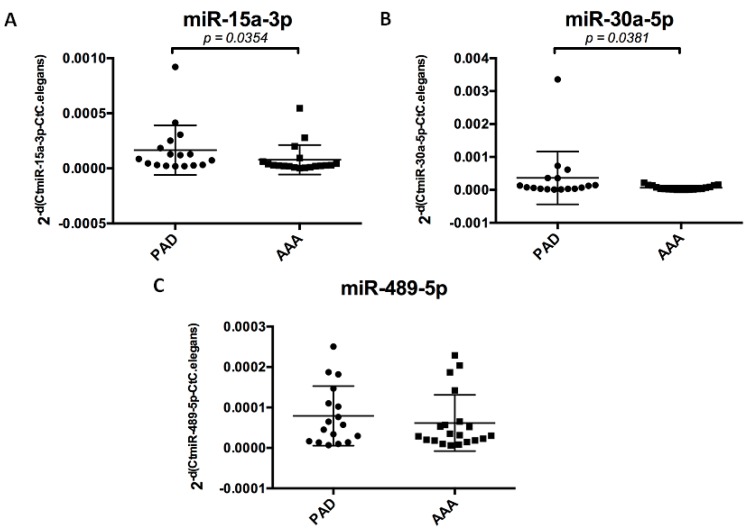
Relative plasma quantification of the three miRNAs (mir-15a-3p (A); miR-30a-5p (B); miR-489-5p (C)) in patients with AAA (*n* = 20) and with PAD without AAA (*n* = 17) by qRT-PCR using the −2ΔΔ*C*_t_ method, with PAD patients as reference and Syn-Cel-miR-39 for calibration. Results are expressed as individual values for each patient in Log (2^−ΔΔ*C*t^) with the mean represented by the horizontal bar.

Expression in the plasma of AAA compared to PAD patients was significantly down-regulated for miR-15a-3p (0.5-fold, *p* = 0.03) and miR-30a-5p (0.8-fold, *p* = 0.04) (Figure 4).

The two miRNAs, miR-15a-3p and miR-30a-5p, significantly modulated in AAA patients are both implicated in the inflammatory response. The expression of miR-30a-5p in plasma is similarly modulated as in ATLOs and M1 macrophages, and those of miR-15a-3p is similarly modulated as in ATLOs and both macrophages. Our findings indicate that miR-15a-3p and miR-30a-5p have potential as biomarkers. Further investigations will be required to evaluate their targets in order to better understand AAA pathophysiology.

## 3. Experimental Section

### 3.1. Human Abdominal Aorta and Plasma Samples

Our study conformed to the principles outlined in the Declaration of Helsinki. We obtained informed consent in writing from each patient undergoing open surgical repair of an infrarenal aneurysm and recovered the biological samples as surgical waste, in accordance with French laws on medical ethics. Thus, samples of human aneurysmal infrarenal aortic walls were obtained from 20 patients in Haulon’s vascular surgery unit (Hôpital Cardiologique, CHRU Lille, France). The samples, each with an intraluminal thrombus, were collected in normal saline solution and transported to the laboratory at 4 °C. Each sample was dissected into transversal slides after orientation of the tissue by macroscopic analysis. Sections were formalin-fixed, paraffin-embedded, and kept at 4 °C or they were snap-frozen in liquid nitrogen, in both cases for further analyses (Table S1).

Healthy non-aneurysmal aortas (control samples) came from deceased patients providing multiple organ retrievals, with the written consent of their families and the authorization of the French Biomedicine Agency (PFS 11-004). Fourteen control abdominal aorta samples were collected, with the collaboration of Pr. Pruvot’s transplant unit (CHRU, Lille, France). All samples were preserved in normal saline solution at 4 °C for less than 10 h prior to dissection and preparation at the laboratory according to the same protocol described above for the aneurysmal samples.

Plasma samples were collected from another AAA population and from control patients with documented peripheral artery disease (PAD). The AAA and PAD-control populations have been previously described [19]. The LILle Aneurysmal Study (LILAS) was a case-control study that enrolled 42 men with either AAA (*n* = 24) or PAD (*n* = 18) who required vascular surgery at the same hospital (Lille, France). Case/AAA patients were eligible if the AAA diameter, measured by abdominal ultrasound, exceeded 50 mm or if it had increased more than 10 mm during the past six months. PAD patients were eligible if PAD was diagnosed in the aortic, iliac, or femoral arteries and AAA had been ruled out by abdominal ultrasound. The hospital’s ethics committee approved both studies, and each patient and subject provided written informed consent.

### 3.2. Histological Analysis and Immunohistochemistry

Histological and immunohistochemical analyses were performed on the 20 paraffin-embedded tissue samples to locate the different inflammatory cells, except for Oil red O analysis, which was done on frozen sections. Immunohistochemical analyses were performed as previously described [10]. We identified inflammatory cells with antibodies specific for each cell type: Neutrophils with mouse anti-CD66e (1/10, Novus, Littleton, CO, USA) antibody, T lymphocytes with mouse anti-CD3 (1/200, DAKO Corporation, Carpenteria, CA, USA) antibody, B lymphocytes with mouse anti-CD20 (1/50, Abcam, Cambridge, UK) antibody and mast cells with mouse anti-mast cell tryptase (1/100, Abcam) antibody. Immunostaining used the appropriate biotinylated secondary antibodies (Table S2, 1/200, Vector laboratories, Burlingame, CA, USA), streptavidin-horseradish peroxidase (Vectastain ABC kit, Vector Laboratories) and the AEC substrate-chromogen system (Sigma, Saint-Louis, MO, USA). Finally, slides were mounted with Glycergel (DAKO Corporation) and analyzed with an Axioplan 2 microscope (Carl Zeiss, Marly Le Roi, France), which includes an HRc camera (AxioVision-Deconvolution 3D, Carl Zeiss). The relative quantification of the thickness and area of ATLO was performed using ImageJ software (Version 1.45, Bethesda, MD, USA).

### 3.3. Laser Capture Microdissection (LCM)

Frozen sections (8-µm) of aneurysmal samples were stained for CD20 to identify the areas abundant in ATLO. Adjacent 18-μm unstained frozen sections were prepared for LCM on PEN membrane glass slides (MDS Analytical Technologies, Sunnyvale, CA, USA) by dehydration in alcohol and then clearance with xylene (Sigma-Aldrich, Saint-Louis, MO, USA). LCM was performed with an ArcturusXT microdissection instrument (MDS Analytical Technologies). Microdissected areas were collected on CapSure Macro LCM Caps (MDS Analytical Technologies), as previously described [10].

### 3.4. RNA Extraction, miRNA Screening and qRT-PCR Analyses

RNA was extracted from 10 mm^2^ of LCM-isolated areas with 200 μL TRI Reagent^®^ (Ambion, Austin, TX, USA), according to the manufacturer’s instructions, and from 200 μL plasma with the miRNeasy Mini Kit (Qiagen, Venlo, The Netherlands), also according to the manufacturer’s instructions. Syn-cel-miR-39 miScript miRNA Mimic (Qiagen) (5 µL) was added to each plasma sample as a spike-in control and used for calibration.

LCM-dissected areas abundant in ATLOs obtained from two different aortic tissue samples were screened for miRNA expression with the human miRNA 8 × 15 k microarray (850 known human miRNAs, according to the Sanger database version 12.0, Agilent Technologies). Tissue samples were analyzed individually on the same microarray. After dephosphorylation and denaturation, the RNA samples were labeled with Cyanine 3-pCp, then purified with micro Bio-Spin 6 chromatography columns (Bio-Rad Laboratories, Hercules, CA, USA). The purified labeled miRNAs were incubated with the array in hybridization chambers (Agilent) for 20 h at 55 °C. The slides were then washed as described in the protocol. The microarray was then run on a SureScan microarray scanner (Agilent Technologies, Santa Clara, CA, USA). Raw data were acquired with Feature Extraction Software (Version 10.7.3.1, Agilent, Santa Clara, CA, USA). Data are available at the Gene Expression Omnibus (GEO) (www.ncbi.nlm.nih.gov/geo) under the accession number GSE63541. We used the R package script *AgiMicroRna* for normalization. The miRNAs detected in ATLOs were selected based on their normalized values calculated with positive and negative controls. We then considered a miRNA detected in ATLOs when associated with a positive normalized value obtained with the 16 probes corresponding to the miRNA for the two samples tested.

We reverse-transcribed 100 ng of total RNA using the miScript II RT kit (Qiagen) and the cDNA was amplified with miScript PreAMP PCR kit (Qiagen). The PCR was then performed with the miScript SYBR Green PCR kit (Qiagen) on a Mx3000P Q-PCR system (Agilent Technologies), according to the manufacturer’s instructions. PCR miScript Primer Assays (Qiagen) for miR-15a-3p (MIMAT0004488), miR-30a-5p (MIMAT0000087), miR-489-3p (MIMAT0002805) and RNU6-2 were used for qRT-PCR.

### 3.5. Statistical Analysis

Statistical analysis was performed with the Mann-Whitney test using GraphPadPrism Software. The Mann-Whitney test was performed to compare plasma samples. A value of *p* < 0.05 was considered statistically significant.

## 4. Conclusions

This study showed the specific distribution of inflammatory cells in human AAA, to characterize B/T cell aggregates organized in lymphoid structures known as ATLOs. Despite limitations due to the small sample size in the array and PCR experiments, the miRNA profiling of isolated ATLOs enabled the detection of 164 miRNAs out of 850 miRNAs screened , and the three miRs with the highest expression (miR-15a-3p, miR-30a-5p and miR-489-3p) were further characterized. With the exception of miR-30a-5p, a similar modulation was found in ATLOs and the two subtypes of macrophages. Plasma modulation of miR-15a-3p and miR-30a-5p found in a relatively small group of AAA patients compared to PAD, underlines the potential of miRNAs isolated from ATLOs to be potential biomarkers of AAA, though the data should be validated in a larger group of patients. Our data emphasize the potential of miR-15a-3p and miR-30a-5p not only as biomarkers of AAA, but also as triggers of ATLO evolution. Further investigations with larger samples sizes will be required to validate our findings and evaluate targets of the identified miRNAs for better understanding of AAA pathophysiology.

## Supplementary Materials

Supplementary materials can be found at http://www.mdpi.com/1422-0067/16/05/11276/s1.
