# Supplementary material for: Adventitial Tertiary Lymphoid Organs as Potential Source of MicroRNA Biomarkers for Abdominal Aortic Aneurysm
Source: Int J Mol Sci. 2015 May 18;16(5):11276–93. doi: 10.3390/ijms160511276 (PMC4463700; doi:10.3390/ijms160511276)
Supplement: Supplementary file 1 [file ijms-16-11276-s001.pdf]

Supplementary Information

Table S1. Use of the AAA tissue samples for the presence of ATLOs by immunohistochemistry and for miRNA analyses.

**Samples Number**	**1**	**2**	**3**	**4**	**5**	**6**	**7**	**8**	**9**	**10**	**11**	**12**	**13**	**14**	**15**	**16**	**17**	**18**	**19**	**20**
**ATLOs**	−	−	−	+	−	+	−	+	−	+	+	+	+	−	+	−	+	−	+	+
**Microarray**	−	−	−	−	−	+	−	−	−	−	−	−	−	−	−	−	−	−	−	+
**RT-PCR**	−	−	−	−	−	+	−	−	−	+	−	+	−	−	−	−	−	−	−	+

Table S2. Primary antibodies used for immunohistochemistry location of inflammatory cells.

**Cell Type**	**Antigen**	**Catalog Number, Company**	**Species**	**Dilution**	**Incubation Length**
M1 macrophages	CD 68 EBM11 Clone	M0718, Dako	Mouse	1/50	2 h
M2 macrophages	CD 206	SC-34577, Santa Cruz	Goat	1/50	20 h
B lymphocytes	CD 20	Abcam	Mouse	1/50	2 h
T lymphocytes	CD 3	M0756, Dako	Mouse	1/50	2 h
Mast cells	Mast cell tryptase	ab2378, Abcam	Mouse	1/50	30 min
Leukocytes	CD 66e	NB100-65728, Novus Biologicals	Mouse	1/10	2 h
